# Cross-national variation in the association between family structure and overweight and obesity: Findings from the Health Behaviour in School-aged children (HBSC) study

**DOI:** 10.1016/j.ssmph.2022.101127

**Published:** 2022-05-26

**Authors:** Anne-Siri Fismen, Otto Robert Frans Smith, Arnfinn Helleve, Ellen Haug, Angeline Chatelan, Colette Kelly, Anna Dzielska, Paola Nardone, Marina Melkumova, Oya Ercan, Jaroslava Kopcakova, Giacomo Lazzeri, Knut-Inge Klepp, Oddrun Samdal

**Affiliations:** a Department of Health Promotion, Centre for Evaluation of Public Health Measures, Division of Mental and Physical Health, Norwegian Institute of Public Health, Bergen, Norway; b Centre for Evaluation of Public Health Measures, Norwegian Institute of Public Health, Oslo, Norway; c Department of Health Promotion and Development, University of Bergen, 5020, Bergen, Norway; d Department of Teacher Education, NLA University College, 5012, Bergen, Norway; e School of Public Health, Université libre de Bruxelles, Route de Lennik 808, 1070, Brussels, Belgium; f Health Promotion Research Centre, National University of Ireland Galway, Ireland; g Department of Child and Adolescent Health, Institute of mother and Child, Warsaw, Poland; h National Centre for Disease Prevention and Health Promotion, Italian National Institute of Health, Rome, Italy; i Arabkir Medical Centre-Institute of Child and Adolescent Health, Yerevan, Armenia; j Department of Pediatrics, Istanbul University-Cerrahpasa, Turkey; k Department of Health Psychology and Research Methodology, Faculty of Medicine, P. J. Safarik University in Kosice, 040 01, Kosice, Slovakia; l Department of Molecular and Developmental Medicine, University of Siena, Siena, Italy; m Division of Mental and Physical Health, Norwegian Institute of Public Health and Department of Nutrition, University of Oslo Oslo, Norway

## Abstract

**Background:**

Trends of increased complexity in family structure have developed alongside increasing prevalence of overweight and obesity. This study examines cross-national variations in the likelihood of living with overweight and obesity among adolescents living with one parent versus two parents, as well as the influence of living with stepparents, grandparents and siblings. Furthermore, the study explores how these associations relate to age, gender and individual-level socioeconomic status (SES) and country-level SES. We hypothesised that adolescents living in one-parent versus two-parents families, were more likely to live with overweight and obesity.

**Methods:**

The study is based on nationally representative data from 41 countries participating in the 2013/14 Health Behaviors in School-Aged Children study (n = 211.798). Multilevel logistic regression analysis was used to examine the associations between family structure and overweight and obesity by age, gender, SES, and geographic region, among adolescents aged 11, 13 and 15 years.

**Results:**

Living with one versus two parent(s) was associated with a higher likelihood of overweight and obesity (OR_adj_.1.13, 95%CI 1.08,1.17). Age, gender, individual-level SES, and living with grandparents were also associated with a higher likelihood of overweight and obesity, whereas living with siblings was associated with a lower likelihood of overweight and obesity. The effect of family structure varied also by age and gender with no significant associations found between living with one parent and overweight and obesity in the 15-year-old age group. Some cross-national variation was observed, and this was partly explained by country-level SES. The effect of family structure increased by a factor 1.08 per one-unit change in country-level SES (OR 1.08, 95%CI1.03, 1.12).

**Conclusion:**

The study indicates that living in a one-parent family, as well as living together with grandparents, are associated with overweight and obesity among adolescents, particularly in the Nordic European region. Existing welfare policies may be insufficient to eliminate inequalities related to family structure differences.

## 1 Introduction

Adolescent overweight and obesity is widely recognized as a public health challenge (WHO, 2014). Surveillance studies indicate that one in five European adolescents live with overweight or obesity (Inchley et al., 2020). Increasing prevalence is shown in one-third of western countries and no country shows a downward trend (Ahluwalia et al., 2015; Inchley et al., 2020). As living with overweight and obesity is associated with a range of physical and psychosocial challenges during adolescence (Lobstein et al., 2004) and increased risk of illness in adulthood (Global et al., 2016; Sommer & Twig, 2018; Weihrauch-Bluher et al., 2019), understanding factors that may influence adolescents’ weight status and identifying possible arenas for preventative actions are important public health priorities.

Ecological models (Sallis et al., 2008) highlight the environmental setting and emphasize the importance of the reciprocal influences of proximal and distal developmental environments on individual outcomes. The family setting is identified as having a core influence on children's health and health behaviors, including weight status (Chai et al., 2019). Modern families are characterized by increased complexity in their compositions. A growing percentage of adolescents live within one-parent families, in joint custody or reconstructed families consisting of one parent and a stepparent (Pearce et al., 2018; Report, 2016; Steinbach, 2019; Milanovi ć et al.). Others live with grandparents (as their main custodians or as co-residence in a multigenerational home) or in foster care (Glaser et al., 2018). As the trend of increased family structure heterogeneity develops alongside trends of high and increasing overweight and obesity rates, evaluating the role of family structure differences is of public health interest. Importantly, family structure and its influence may represent a dimension of social inequality relevant for research and policy actions.

A systematic review concluded that living in a one-parent versus two-parents family was associated with a higher risk of obesity among preschool and primary school-aged children (Duriancik & Goff, 2019). Studies investigating family structure differences in adolescents' weight status produced mix results (Chen & Escarce, 2014; Eidsdottir et al., 2013; Parikka et al., 2015; Tabak et al., 2012). It remains unsure if living in a one-parent family is associated with a higher likelihood of overweight and obesity among adolescents, particularly in the European context and with a cross-national perspective, and more research is needed to explore this association. Adolescents growing up in one-parent families may experience economic barriers to engaging in healthy behaviors, as the family may have a lower income (Moncrief et al., 2014; Youngblut et al., 2005), which may challenge a parent's ability to overcome financial barriers related to a healthy lifestyle, e.g. purchase of healthy food (Berge et al., 2013) and equipment and access to physical activity classes or events. Similarly, also the available time to prepare healthy meals (Berge et al., 2013) and engage or accompany their child to sporting activities (Badura et al., 2021; Langoy et al., 2019) may vary across one-parent versus two-parent families. The relevance of these factors is underlined by adolescents participating in the project “CO-CREATE – Confronting obesity, co-creating policy with youth”, in which lower household income, less time for food preparation and less availability of home cooked meals were mentioned among the drivers of adolescent obesity (Savona et al., 2021). Furthermore, children living in one-parent families may experience less stringent rules with regard to sedentary behaviours than do children living in two-parent families (Stahlmann et al., 2020).

Concurrent with the substantial income decline that may follow a divorce or breakup, one-parent families may experience increased psychosocial stress and financial strain from heavy workloads and relocation to more disadvantaged neighbourhoods (Flouri et al., 2016). These mechanisms may overlap with those relevant for low socioeconomic status (SES), which is a well-known risk factor for overweight and obesity (Vazquez & Cubbin, 2020). Therefore, it is important to determine if there is an independent association between family structure and overweight and obesity or if family structure is merely a proxy for SES. Further, previous studies of family structure differences have almost exclusively defined family structure as simply one- or two-parent families (Duriancik & Goff, 2019; Troxel et al., 2014; Zaborskis et al., 2020), and thus ignored the possible impact of living in more complex family compositions consisting of stepparents and grandparents. Previous research link grandparental co-residence with increased risk of obesity among children (An et al., 2020), but few studies have examined such associations among adolescents, particularly in Europe. More nuanced analysis of family structure differences, including the role of several family members living together with adolescents (stepparents, grandparents, siblings) may provide a deeper understanding of contextual determinants related to adolescents' weight status. Of note, one study (Fismen et al., 2020) reported less favorable food habits among adolescents living in a one-parent family as well as in a stepfamily (mother and stepfather), compared to a two-parent family, and this relationship was strongest among those living in a stepfamily. This underlines that the number of adults in the family does not itself explain differences in adolescents' health behaviors, and that psychosocial conditions like e.g. family cohesion and family climate should be considered in terms of family structure differences. Another aspect is that the effect of family structure heterogeneity may be mediated by policy actions and sociocultural factors (Zaborskis et al., 2020). Family-related policies are central to most countries’ welfare programs, with substantial investment seen in many countries. Thus, adding a cross-national perspective may be particularly valuable and provide clinicians, policy makers and health program developers with a better understanding of how to target overweight and obesity interventions in the adolescent population.

The present study aims to examine associations between family structure and overweight and obesity (assessed by BMI-z-score for age and sex) in a nationally representative sample of adolescents aged 11, 13 and 15 years, from Europe and Canada participating in the 2013/2014 “Health Behaviour in School-aged Children study. A WHO Cross-national study” (HBSC). To the best of our knowledge, this is the first time such associations are studied in a large, cross-national sample of adolescents. Based on existing research on associations between family structure and health and health behaviours, we hypothesise that adolescents living in one-parent are more likely to live with overweight and obesity, compared to adolescents living in two-parents families.

## 2 Method

### 2.1 Study design and data collection

The present study is based on nationally representative data from adolescents aged 11, 13, and 15 years from 41 countries participating in the cross-national Health Behavior in School-aged Children (HBSC, www.hbsc.org) survey in 2013/2014. The overall aim of the HBSC study is to enhance the understanding of young people's health behaviors in their social settings. The students answered a standardized questionnaire at school after receiving instructions from their teacher. Oral and written information on the confidentiality of their responses were provided and participation was confidential and voluntary. Most countries used school class as the primary sampling unit (some countries used schools as the sampling unit). Schools/classes that declined to participate, as well as students absent on the day the survey was carried out, were the two main sources of non-response and were not followed up. In the majority of the countries included, response rates at the school, class, or student level exceeded 80% (Inchley et al., 2013).

Ethical consent from the institutional ethics committee(s) or any relevant board at country or regional level was required. The HBSC Data Management Centre checked the quality of the data collected, performed appropriate cleaning of the data and merged national data sets into an international data file. The methodology for data collection is described in the HBSC protocol (Currie et al., 2014), which prescribes consistency in sampling plans, survey instruments and data collection. Detailed information about the study is available at http://www.hbsc.org/

### 2.2 Measures

**Family structure** was measured by one item “Please answer this first question for the home where you live all or most of the time and tick the people who live there”. The response categories were: mother, father, stepmother (or father's partner), stepfather (or mother's partner), grandfather, grandmother, foster home, and others. The data was coded into two categories: one parent in the main home, two parents in the main home. Participants with no parents in the main home (1.8%) were excluded from the analysis. To further describe family structure differences, binary variables were derived for stepparent in the main home (yes/no) and grandparent(s) in the main home (yes/no) for all students. Similarly, having siblings in the main home (yes/no) was derived from two items referring to where the respondent lived all or most of the time: “Please indicate how many brothers and sisters live here (including half, step or foster brothers and sisters)” “How many brothers?” and “How many sisters?”.

**Overweight and obesity** was based on self-reported weight and height measured by the questions: “How much do you weigh without clothes?” and “How tall are you without shoes?” BMI (in kg/m^2^) was calculated and classified into “overweight” and “obesity” based on the well-established international standardized age- and sex-specific cut-off points proposed by Cole and Lobstein (Cole & Lobstein, 2012) for the International Obesity Task Force (IOTF). Because of the low prevalence of obesity (2.6%), overweight and obesity were combined into one weight status category: overweight and obesity.

Validation studies of self-reported height and weight suggest that boys tend to underestimate weight and that both genders tend overestimate height (Perez et al., 2015). However, self-reported measures of height and weight are considered suitable measures for identifying valid relationships in epidemiological studies (Aasvee et al., 2015; Goodman et al., 2000; Spencer et al., 2002).

**Socioeconomic status** was assessed using the family affluence scale (FAS) (Hartley et al., 2016), which is considered a valid SES-indicator (Hobza et al., 2017) also for cross-national comparison (Boyce et al., 2006).FAS is a measure of material affluence derived from the characteristics of the family's household and consists of six items (family car, number of computers, own bedroom, family holidays, number of bathrooms, dishwasher in home). Each student was assigned an individual FAS score (individual-level SES) ranging from 0 to 13 and each country a mean FAS score (country-level SES), which was calculated from individual FAS within the respective country. Individual-level SES, as well as country-level SES, were included in the analysis.

**Country classifications:** European sub-regions were coded according to the EuroVoc classification (Union, 2020), which encompasses four separate regions; Northern, Western, Southern and Central Europe. Canada was included in the Western European group and Israel in the Southern European group.

### 2.3 Statistics

Multilevel logistic regression analysis (binomial distribution, logit link) was used to examine the associations between overweight and obesity (dependent variable) and family structure. Level-1 units were students, and level-2 units were classes. All countries were pooled together for analysis and the country variable was modelled as a fixed effect. We started with a simple random intercept model with family structure as the only independent variable (model 1). In the next steps, all level-1 predictors (e.g. gender, age, individual-level SES) were first added as fixed main effects (model 2), followed by a model that included the 2-way interactions with the family structure (e.g. family structure*gender). Only interactions that were statistically significant based on the Wald-test, were retained (model 3).

To examine whether potential country variations in the association between overweight and obesity and family structure could be explained by geographical region or country-level SES, the country by family structure interaction was replaced by the two cross-level interactions that included the mentioned country-level variables and family structure (M ö hring, 2012). Geographical region was added first (model 4a), followed by an analysis with both the geographical region and country-level SES (model 4b).

Individual-level SES (level-1) was group-mean centred to remove potential country-level influences on the estimate of the association between overweight and obesity and individual-level SES (i.e. each student's SES was deviated around their country-mean SES). Country-level SES was grand-mean centred to ease interpretation (i.e. each country's SES was deviated around the overall mean SES).

All analyses were conducted in STATA v.15.

## 3 Results

The current sample included 211.798 students (49% boys) from 41 countries. Boys were underrepresented in the Irish (39%) and the Russian sample (44%). Table 1 reports cross-country heterogeneity in family structure, SES and prevalence of overweight and obesity. The percentage of adolescents living with one parent ranged from 6% in Albania to 38% in Greenland. In the total sample, one-fourth lived with one parent and three quarters lived with two parents. Almost one out of ten lived with a stepparent (and one of their parents) and one out of six had grandparents in the main home. Four out of six had siblings in the main home. The percentage of adolescents living with overweight and obesity was 15% in the total sample and ranged from 8% in Denmark to 27% in Malta. Overall, higher percentages of adolescents living with overweight and obesity were observed in the Southern European region, while lower percentages were observed in the Northern European region. Some countries had high rates of missing data on BMI. The country-level mean SES (group-mean centred FAS) varied from 4.9 (Albania) to 9.9 (Luxemburg). The Netherlands was excluded due to missing data on siblings.

**Table 1:** Characteristics of the study population (n = 211,798 students).

	N	Boys (%)	13 yr (%)	15 yr (%)	Country-level SES	One parent in the main home (%)	Stepparent in the main home (%)	Grand parent(s) in the main home (%)	Sibling(s) in the main home (%)	Overweight and obesity (%)	Missing BMI scores (%)
**Western Europe**											
Austria	3416	46.5	31.7	37.0	9.0	22.5	7.6	20.0	89.2	13.2	10.2
Belgium (French)	5814	49.7	33.7	32.7	8.5	26.9	16.9	6.3	91.9	13.7	36.0
Belgium (Flandern)	4359	54.9	27.0	39.5	8.9	23.1	14.5	14.2	89.8	12.0	14.4
Canada	12530	49.5	37.3	38.5	8.7	28.5	11.2	6.1	86.0	24.8	24.6
Switzerland	6592	49.5	36.0	33.9	9.6	20.2	8.1	8.3	90.8	10.3	13.5
Germany	5893	50.9	35.1	35.6	8.9	23.4	9.9	14.4	86.0	13.0	18.5
England	5264	51.9	29.9	30.4	8.8	27.6	11.3	8.1	90.4	15.0	56.2
France	5627	50.4	38.6	30.9	8.8	27.5	13.1	6.7	90.9	11.6	14.6
Ireland	4064	38.9	36.8	37.3	8.7	21.1	6.3	6.9	92.8	15.2	71.9
Luxembourg	3259	47.4	36.2	34.6	9.9	25.8	12.1	6.8	88.7	14.2	17.0
Scotland	5806	50.3	35.4	32.4	8.8	31.4	11.8	4.9	89.8	15.0	71.2
Wales	5041	51.0	36.5	27.7	9.1	35.5	11.8	4.2	86.1	19.3	59.4
**Central Europa**											
Albania	5011	49.0	33.1	34.5	4.9	6.4	0.8	39.1	95.6	9.6	11.6
Armenia	3640	47.7	31.7	28.5	5.3	9.5	0.1	51.7	97.6	14.3	26.2
Bulgaria	4586	52.1	32.3	34.4	6.8	21.0	5.1	34.5	57.8	18.1	8.3
Czech Republic	4999	47.6	34.0	34.9	8.0	29.4	12.0	23.6	86.8	14.7	7.1
Croatia	5696	50.2	34.9	33.8	7.2	14.1	4.2	33.9	88.4	16.0	4.7
Hungary	3845	49.6	34.8	28.2	6.4	28.5	10.2	13.8	85.2	16.1	13.2
Republic of Moldova	4472	50.8	33.3	33.3	5.3	19.3	4.7	37.8	84.4	10.6	0.2
North-Macedonia	4137	49.8	31.5	35.0	6.9	10.9	0.6	48.7	100.0	18.7	20.0
Poland	4475	49.7	33.6	32.8	6.9	20.6	6.6	23.5	84.3	15.2	8.6
Romania	3824	47.4	31.4	36.8	5.6	20.8	3.9	25.8	76.6	14.5	39.5
Russian federation	4616	43.8	38.2	31.5	6.2	29.7	10.6	25.5	85.2	13.7	13.7
Slovenia	4950	48.8	34.8	32.5	9.0	18.0	6.1	30.4	87.6	17.3	5.5
Slovakia	6076	50.3	40.2	30.6	7.2	22.4	0.9	0.8	86.8	14.5	11.6
Ukraine	4466	47.4	30.5	36.9	5.3	25.6	8.4	34.4	70.1	9.9	7.7
**Northern Europe**											
Denmark	3867	46.8	35.3	32.9	9.2	24.2	10.1	1.2	93.6	8.3	10.4
Estonia	3980	50.3	35.3	31.1	7.5	32.1	14.0	16.5	84.7	14.4	10.7
Finland	5878	49.2	32.4	33.7	8.4	24.4	14.5	4.5	94.2	15.5	11.3
Greenland	927	47.6	36.8	30.7	5.8	38.3	15.2	8.9	90.8	20.4	45.4
Iceland	10490	50.0	35.3	31.7	8.5	27.7	13.5	2.7	91.1	15.2	20.0
Lithuania	5578	50.8	35.3	29.4	8.1	27.0	8.9	24.4	86.4	11.6	31.6
Latvia	5375	47.8	35.2	31.0	6.5	32.6	11.5	22.2	78.7	15.1	4.0
Norway	3380	48.7	30.8	28.8	9.8	19.9	9.8	4.6	93.1	10.4	14.2
Sweden	7515	49.6	29.6	36.3	9.2	28.2	10.7	2.1	93.2	13.6	17.4
**Southern Europe**											
Greece	4098	49.7	35.0	32.0	6.6	14.0	3.5	17.5	86.3	20.9	4.1
Israel	6148	48.6	30.1	30.1	7.7	13.3	3.6	6.0	100.0	14.4	31.4
Italy	4024	50.3	35.1	31.6	7.5	15.7	3.3	16.9	86.3	17.8	14.7
Malta	2214	51.4	35.8	28.2	9.2	11.8	1.4	2.7	78.4	26.8	45.2
Portugal	4910	47.4	39.8	27.1	8.5	24.0	8.8	14.6	77.3	18.1	2.5
Spain	10956	49.2	38.8	33.9	8.2	18.5	5.6	10.8	5.3	17.1	14.1
Total	211798	49.2	34.6	33.1	7.9	23.2	8.7	15.6	83.2	15.1	20.5
%missing	–	0	0.8		8.3	4.6	4.6	4.6	5.7	20.5	

### 3.1 Family structure differences in overweight and obesity

As shown in Table 2, adolescents living with one parent were more likely to live with overweight and obesity than were their counterparts living with two parents (crude association OR 1.17, 95%CI 1.13, 1.21, model 1). Living with a stepparent (one parent + stepparent in the main home) was not significantly associated with increased/decreased likelihood of overweight and obesity. Having grandparent(s) in the main home was associated with a higher OR of overweight and obesity (OR 1.19, 95%CI 1.14, 1.24, model 2). Having siblings in the main home (OR 0.79, 95%CI 0.76, 0.83), and higher individual-level SES (OR 0.94, 95%CI 0.94, 0.95) were associated with a lower OR likelihood of overweight and obesity.

**Table 2:** Crude and adjusted models for the associations between family structure and adolescents’ overweight and obesity, all countries.

	Model 1		Model 2				Model 3				Model 4b			
	OR	95% CI	OR		95% CI		OR		95% CI		OR		95% CI	
Overweight and obesity														
One parent in the main home	1.17***	[1.13,1.21]		1.13***		[1.08,1.17]		1.34		[0.88,2.06]		1.30***		[1.17,1.45]
Boys				1.67***		[1.62,1.73]		1.71***		[1.65,1.77]		1.71***		[1.64,1.77]
13 yr olds				0.96*		[0.92,1.00]		0.98		[0.93,1.03]		0.98		[0.93,1.03]
15 yr olds				0.98		[0.94,1.02]		1.01		[0.97,1.06]		1.01		[0.96,1.06]
Individual-level SES				0.94***		[0.94,0.95]		0.94***		[0.94,0.95]		0.94***		[0.94,0.95]
Stepparent in the main home				0.97		[0.91,1.03]		0.96		[0.91,1.02]		0.96		[0.90,1.02]
Grandparent(s) in the main home				1.19***		[1.14,1.24]		1.20***		[1.15,1.25]		1.20***		[1.15,1.25]
Siblings in the main home				0.79***		[0.76,0.83]		0.79***		[0.75,0.82]		0.79***		[0.75,0.82]
One parent x boys								0.93*		[0.86,0.99]		0.93*		[0.86,0.99]
One parent x 13 yr								0.89*		[0.82,0.98]		0.90*		[0.82,0.98]
One parent x 15 yr								0.85***		[0.78,0.93]		0.86***		[0.79,0.94]
One parent x country-level SES												1.08***		[1.03,1.12]
One parent x Northern Europe												1.01		[0.91,1.11]
One parent x Southern Europe												0.93		[0.82,1.05]
One parent x Eastern Europe												0.99		[0.87,1.12]
Constant	0.10***	[0.09,0.11]		0.09***		[0.08,0.10]		0.08***		[0.07,0.10]		0.08***		[0.07,0.10]
Variance estimates														
Random intercept	0.18***	[0.16,0.21]		0.17***		[0.14,0.19]		0.17***		[0.14,0.19]		0.17***		[0.14,0.19]

Reference categories: gender; girls, age; 11 year olds, family structure; two parents in the main home, region; Western Europe. Country fixed effects are not shown for models 1–4. Country x one parent in main home interactions are not shown for model 3.

Significant interaction effects are shown in Table 2. The effect of living with one versus two parents on overweight and obesity was less pronounced in boys than girls, and less pronounced in older adolescents (13- and 15-years-old) compared to younger adolescents (11-year-olds, model 3). Wald test for gender interaction was as follows χ^2^ = 5.06, *p* = .02 and for age interactions: χ^2^ = 12.19, *p* = .002. The model derived conditional odds ratio for boys (OR = 1.09, 95%CI 1.03, 1.14) was lower than for girls (OR = 1.17, 95%CI 1.11, 1.24), whereas the conditional odds ratio's for 13- and 15-year olds (OR = 1.11, 95%CI 1.04, 1.18; OR = 1.06, 95%CI 1.00, 1.13, ns) were lower than for 11-year olds (OR = 1.24, 95%CI 1.16, 1.33). The 2-way interactions between family structure and individual-level SES (χ^2^ = 1.20, *p* = .27), living with a stepparent in the main home (χ^2^ (WHO, 2014) = 0.02, *p* = .90), having grandparent(s) in the main home (χ^2^ = 0.96, *p* = .33), and having sibling(s) in the main home (χ^2^ = 0.00, *p* = .96) were all not statistically significant and were therefore not included in subsequent models.

### 3.2 Cross-national differences

**Fig. 1:**
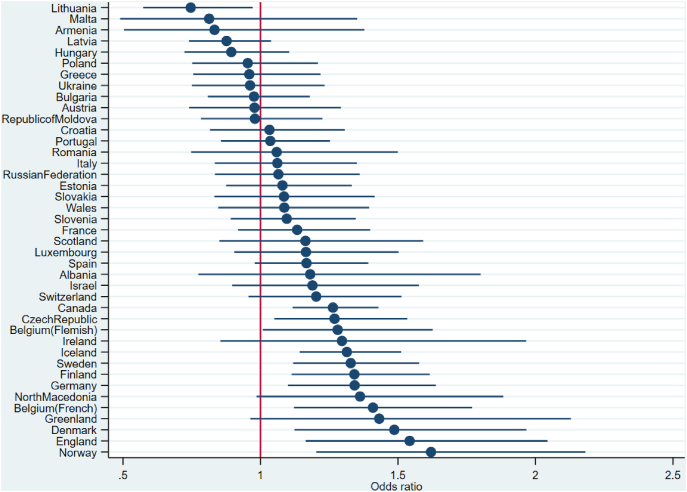
Associations between living with one versus two parent(s) and overweight and obesity across countries, adjusted for co-variates (estimates derived from model 3).

As shown in Fig. 1 (conditional OR), both the strength and the direction of the association varied by country. The strongest association between living with one parent and a higher OR of overweight and obesity was identified in the Norwegian sample (OR 1.61, 95%CI 1.20, 2.17). In one country – Lithuania – living with one parent was significantly associated with a lower OR of overweight and obesity (OR 0.74, 95%CI 0.57, 0.97).

**Fig. 2:**
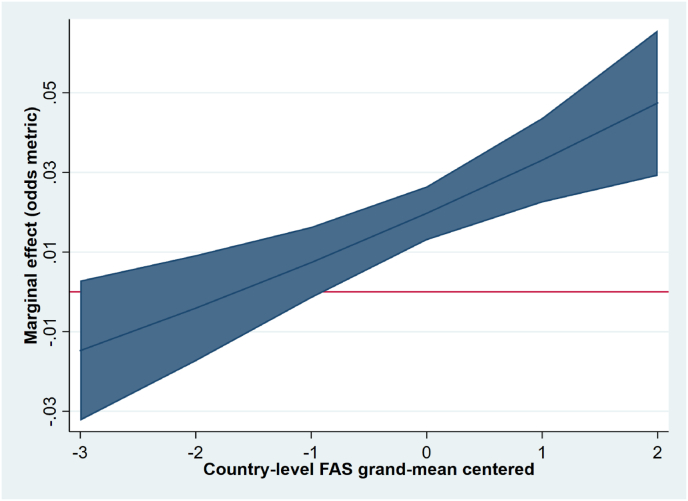
Conditional marginal effects of living with one versus two parent(s) by country-level SES (estimates derived from model 4b).

Cross-national variation in the relationship between living with one parent and overweight and obesity was partly explained by geographical region, with weaker associations between adolescents living with one parent and overweight and obesity demonstrated in Eastern Europe (OR 0.85, 95%CI 0.77, 0.93 model 4a, not shown in Table 2), and Southern Europe (OR 0.86, 95%CI 0.77, 0.97), as compared to Western Europe (reference category). Northern Europe did not differ from Western Europe (OR 0.95, 95%CI 0.87, 1.04) in this regard. After adding country-level SES to the model, the interaction between SES and geographical region became non-significant, suggesting that this association could largely be explained by differences in country-level SES between regions (OR 1.08, 95%CI 1.03, 1.12, model 4b, see Table 2). As shown in Fig. 2, the effect of family structure was stronger with increasing country-level SES scores. For example, the conditional OR of family structure in countries with low country-level SES (−3) was 0.90 (ns), whereas the conditional OR was 1.31 (*p* < .05) in countries with high country-level SES (+2).

## 4 Discussion

This is one of few studies reporting on family structure differences in adolescent overweight and obesity and is thus a step forward in filling a gap in mapping the social determinants of health and health inequalities in adolescence. Overall, adolescents living with one parent were more likely to live with overweight and obesity than were their counterparts living with two parents. Substantial cross-country variation was observed, with stronger associations identified in countries with high, compared to low, country-level SES.

### 4.1 Associations between family structure and overweight and obesity

The findings of a higher OR of overweight and obesity among adolescents living with one versus two parent(s) are in line with two previous studies (Chen & Escarce, 2014; Tabak et al., 2012) but contrast other (Eidsdottir et al., 2013; Parikka et al., 2015). The findings also correspond with a current review of family structure differences in overweight and obesity among preschool and primary school age children (Duriancik & Goff, 2019). Higher OR of overweight and obesity among adolescents living with one parent may reflect an interplay between several factors relevant for weight status. Most likely, family structure exerts an indirect effect on overweight and obesity through related differences in energy balance-related behaviours (EBRB). The present findings should be viewed in light of previous studies. It has been suggested that living with one versus two parent(s) s were associated with less healthy food habits (Baek et al., 2014; Elfhag & Rasmussen, 2008; Fismen et al., 2020; Renzaho et al., 2014; Stewart & Menning, 2009; Zaborskis et al., 2020), less regular meal frequency (Franko et al., 2008; Larson et al., 2013; Lazzeri et al., 2016; Levin et al., 2012; Pearson et al., 2010; Sharif et al., 2017; Stewart & Menning, 2009), lower levels of physical activity (Langoy et al., 2019), higher levels of sedentary behaviour (Stahlmann et al., 2020), and higher levels of sleep problems (Troxel et al., 2014), all correlates of overweight and obesity (Al-Khudairy et al., 2017; Fatima et al., 2015). The relationship between EBRB and family structure may relate to differences in time and financial means as well differences in neighbourhood, as those living in deprived areas may have limited choice of healthy foods, due to their lower availability and higher prices (Berge et al., 2013; Moncrief et al., 2014; Thomson & McLanahan, 2012; Youngblut et al., 2005), as well as reduced opportunities for physical activity due to limited walkability in some neighbourhoods. Previous research reported that the relationship between family structure and EBRB is mediated by SES (Zaborskis et al., 2020). However, the present study suggests that family structure differences persist after adjusting for family affluence and that individual-level SES represents only some of the observed family structure differences in adolescents’ overweight and obesity. Other possible underlying mechanisms include time constraints within one-parent families as well as differences related to social structure and routines for family meals, sleep and physical activity levels (Berge et al., 2013; Langoy et al., 2019; Moncrief et al., 2014; Youngblut et al., 2005). These perspectives were not examined in the current study and should be followed up in the future.

Another perspective is that family structure heterogeneity may supersede the structure itself, through underlying processes such as family cohesion and family climate (Covington et al., 2021; Herke et al., 2020; TM, 2012) which may influence adolescent EBRB and consequently their weight status. In the case of separated parents, lower levels of resilience, internalizing and externalizing problems due to the possible presence of interparental conflict and risk behaviours due to inconsistency in parenting styles may be related to inequalities in EBRB (Nunes-Costa et al., 2009; Schaan & Vogele, 2016; van Dijk et al., 2020). Also mental challenges important for weight development, e.g. coping with traumatic experiences, including separation/divorce or loss of parents may be related to emotional or restrained eating (Thomas et al., 2020) as well as higher BMI (Davis et al., 2019). Additionally, parent mental health may come into play; different types of parental stress, e.g. stress associated with divorce and breakups, are linked to increased risk of children's overweight and obesity (Jang et al., 2019). Further, maternal anxiety is associated with children's overweight and obesity among single mothers, but not among married/cohabiting mothers (Belcher et al., 2019). This underlines the need for identifying contextual factors to enhance our understanding of the relationship between mental health and adolescent overweight and obesity and to inform the development of interventions to improve adolescent health outcomes.

Having a stepparent in the main home was not associated with OR for overweight and obesity. This may be surprising as one could expect that stepparents contribute with time and economic resources, as well as with consistency around meals and physical activity – both for healthy content and regularity, which potentially could reduce the likelihood of adolescent overweight and obesity. The finding may underline the importance of family cohesion and family climate. Previous studies have suggested that stepparents may underinvest in non-biological children, because they may be providing resources to their biological children in other households or because they are less committed to non-biological children (Hofferth, 2006). Furthermore, reconstituted families consisting of one parent and a stepparent may be more likely to have one or more strained parent–child relationships, which may influence family diets (Franko et al., 2008).

Having siblings (lower OR) and grandparents (higher OR) in the main home were associated with adolescent weight status. Having siblings has previously been associated with higher levels of physical activity and healthier food habits (Meller et al., 2018), and may explain the observed lower OR of overweight and obesity. Moreover, families with one child are more likely to be child-centred (Khadaroo, 2021), and studies show that permissive parenting is associated with higher BMI (Shloim et al., 2015), and may be more likely to use sedentary forms of child entertainment to get other responsibilities done (Chen & Escarce, 2014). The role of co-habiting grandparents may be viewed in light of mechanisms related to weight perceptions and feeding practices. Grandparents may perceive higher BMI in their grandchildren as an indicator of good nutrition and good health, or provide them with unhealthy food (e.g., sweets and fried food) (An et al., 2020), maybe as an expression of love and kindness (Li et al., 2015). The role of grandparents may be highly relevant in countries where multigenerational households are common. On this background, one could expect higher rates of overweight and obesity in countries with a high percentage of adolescents having grandparents in the main home, e.g. in the Central European region. This was not the case in the current sample. However, the present study provides cross-country comparisons of relative associations between family structure and weight status and does not analyze absolute differences. Further, there may be characteristics of multigenerational families that were not recognized in the current study and which may vary across countries. E.g. while grandparent co-residence may be linked to financial strain or lower SES in some countries, cultural norms may be the underlying mechanism behind multigenerational households in other countries.

The strength of the associations between one-parent families and adolescents living with overweight and obesity decreased with increased age, with no significant associations identified among 15-year-olds. The findings may be considered in relation to middle adolescence (age 15–17 years) as a developmental period characterized by increasing autonomy, greater independence, and less parental influence. Further, the prevalence of overweight and obesity decreased with increased age, which may potentially influence this relationship. The findings underline that family-based interventions addressing overweight and obesity in this age group should be supplemented with interventions in other settings. Interventions targeting adolescents in places outside home, e.g. in schools and arenas for leisure time, accompanied with population-based structural policies addressing EBRB of the whole population, may be more effective.

### 4.2 Cross-national variation

A tendency of a higher likelihood of overweight and obesity among adolescents living with one versus two parent(s) was identified in the majority of the countries examined, with significant associations found in one-fourth. Stronger associations were seen in Northern/Western European countries, explained by high country-level SES. The findings are interesting as countries with high country-level SES are, to a great extent, more egalitarian societies, with family policies and welfare benefits for one-parent families well established (Breivik & Olweus, 2006). Examples are “Welfare-to-work” interventions involving financial sanctions and incentives, training, childcare subsidies and lifetime limits on benefit receipt, which have been used to support or mandate employment among one-parent families (Gibson et al., 2018). The situation around one-parent families has improved in recent years, but they are still in an unfavorable situation when it comes to financial strain and poverty, partly because their employment is more likely part-time and based on temporary contracts (Gibson et al., 2018; Parlament, 2020). Welfare policies in the Northern/Western countries may contribute to a reduction of inequalities in employment status and material resources but may– as indicated by the present results - be insufficient to eliminate inequalities related to family structure differences, particularly in the Northern/Western regions. The weaker association between family structure and overweight and obesity in Southern/Eastern regions may be considered in light of family-based welfare arrangements and grandparental co-residence which may buffer one-parent families' financial strain and lack of time for food preparation and for following up on children's physical activities.

Another perspective is that the Northern/Western European countries have implemented a range of universal overweight and obesity and obesity prevention initiatives (Panter et al., 2018) that support healthy behaviours which could impact weight status. This may explain why the percentage of overweight and obesity was lower in Northern/Western countries compared to other countries. Greater support and engagement in healthy lifestyles in the population as a whole may lead to greater sensitivity of the relative influence of family structure heterogeneity on adolescents’ overweight and obesity rates (Fuller et al., 2019; Gray et al., 2018), which underlines the importance of structural policy actions to prevent overweight and obesity. Studies evaluating absolute differences could possibly provide a better understanding of this perspective.

Previous studies have mainly focused on differences in financial means and available time as pathways to family structure differences in overweight and obesity (Duriancik & Goff, 2019). However, the present study indicate that family structure differences should be considered in a broader context. A more nuanced approach is needed to analyze the circumstances that are particularly detrimental to ensure that the resources needed can attenuate the impact of family structure differences. This may include mental health and psychosocial perspectives. More research is needed to better understand the observed cross-national differences. In particular, the influence of welfare policies and structural policy actions on adolescent overweight and obesity need to be evaluated.

### 4.3 Strengths and limitations

Important strengths of the present study are the large dataset based on nationally representative sampling, as well as standardized measurements across countries. Limitations include the use of self-reported height and weight, which may result in misclassification of BMI due to underestimation of weight among both genders and overestimation of height among boys (Perez et al., 2015). Further, missing BMI data was high in several countries, which may lead to underestimated associations between family structure and overweight and obesity.

SES was measured by FAS, which is an indicator of material affluence (Hartley et al., 2016). The associations between SES and family structure differences may be different if replacing FAS with another SES-indicator. Of note, a recent Swedish study reported that FAS was moderately correlated with parental earned income, and weakly correlated with parent's occupational status (Corell et al., 2021).

In many countries, particularly in the North-Western European countries, common practice is that both parents continue to be involved in the care for their children after divorce or separation. In the current study, we were not able to differentiate between those living in a one-parent family with no involvement from the other parent, and those living most of the time with e.g. mother and part-time with father. Also, it should be noted that some children may have always been reared by one parent so no-one is in effect missing. The results should thus be interpreted considering these limitations.

## 5 Conclusion

Overall, the study provides insights on family structure differences, suggesting that adolescents living in one-parent families and those having grandparents in the main home have a higher likelihood of living with overweight and obesity. It is likely that welfare policies contribute to a reduction of differences in socioeconomic resources but these may be insufficient to eliminate inequities related to family structure differences. A viable research opportunity exists to move forward in a more in-depth analysis of the relevant factors associated with adolescent weight status. Policy actions should support low-income families, families with limited time and poor access to high quality affordable food as well as physical activities during leisure time, but also address different types of stress and psychosocial challenges relevant for one-parent families.

## Conflict of interest

None.

## Author statement

ASF designed the study and drafted the manuscript. ORFS and ASF performed the analysis. All authors contributed with scientific inputs and reviewed/edited the manuscript.

## Ethical statement

This study was conducted according to the guidelines laid down in the Declaration of Helsinki and all procedures involving research study participants were approved by the Ethical Committee in the respective countries included (41 countries).

## Funding

The CO-CREATE project has received funding from the 10.13039/100010661European Union’s Horizon 2020 research and innovation program under grant agreement No 774210. The content of this article reflects only the authors' views and the European Commission is not liable for any use that may be made of the information it contains.

## References

[bib1] Aasvee K., Rasmussen M., Kelly C., Kurvinen E., Giacchi M.V., Ahluwalia N. (2015). Validity of self-reported height and weight for estimating prevalence of overweight among Estonian adolescents: The health behaviour in school-aged children study. BMC Research Notes.

[bib2] Ahluwalia N., Dalmasso P., Rasmussen M., Lipsky L., Currie C., Haug E. (2015). Trends in overweight prevalence among 11-, 13- and 15-year-olds in 25 countries in Europe, Canada and USA from 2002 to 2010. The European Journal of Public Health.

[bib3] Al-Khudairy L., Loveman E., Colquitt J.L., Mead E., Johnson R.E., Fraser H. (2017). Diet, physical activity and behavioural interventions for the treatment of overweight or obese adolescents aged 12 to 17 years. Cochrane Database of Systematic Reviews.

[bib4] An R., Xiang X., Xu N., Shen J. (2020). Influence of grandparental child care on childhood obesity: A systematic review and meta-analysis. Childhood Obesity.

[bib5] Badura P., Hamrik Z., Dierckens M., Gobina I., Malinowska-Cieslik M., Furstova J. (2021). After the bell: Adolescents' organised leisure-time activities and well-being in the context of social and socioeconomic inequalities. Journal of Epidemiology & Community Health.

[bib6] Baek Y.J., Paik H.Y., Shim J.E. (2014). Association between family structure and food group intake in children. Nutrition Research and Practice.

[bib7] Belcher B.R., Maher J.P., Lopez N.V., Margolin G., Leventhal A.M., Ra C.K. (2019). Dual versus single parental households and differences in maternal mental health and child's overweight/obesity. Maternal and Child Health Journal.

[bib8] Berge J.M., Hoppmann C., Hanson C., Neumark-Sztainer D. (2013). Perspectives about family meals from single-headed and dual-headed households: A qualitative analysis. Journal of the Academy of Nutrition and Dietetics.

[bib9] Boyce W., Torsheim T., Currie C., Zambon A. (2006). The family affluence scale as a measure of national wealth: Validation of an adolescent self-report measure. Social Indicators Research.

[bib10] Breivik K., Olweus D. (2006). Children of divorce in a Scandinavian welfare state: Are they less affected than US children?. Scandinavian Journal of Psychology.

[bib11] Chai L.K., Collins C., May C., Brain K., Wong See D., Burrows T. (2019). Effectiveness of family-based weight management interventions for children with overweight and obesity: An umbrella review. JBI Database System Rev Implement Rep.

[bib12] Chen A.Y., Escarce J.J. (2014). Family structure and childhood obesity: An analysis through 8th grade. Maternal and Child Health Journal.

[bib13] Cole T.J., Lobstein T. (2012). Extended international (IOTF) body mass index cut-offs for thinness, overweight and obesity. Pediatric Obesity.

[bib14] Corell M., Chen Y., Friberg P., Petzold M., Lofstedt P. (2021). Does the family affluence scale reflect actual parental earned income, level of education and occupational status? A validation study using register data in Sweden. BMC Public Health.

[bib15] Covington L.B., Patterson F., Hale L.E., Teti D.M., Cordova A., Mayberry S. (2021). The contributory role of the family context in early childhood sleep health: A systematic review. Sleep Health.

[bib16] Currie C.I.J., Molcho M., Lenzi M., Veselska Z., Wild F. (2014). Health behaviour in school-aged children (HBSC) study protocol: Background, methodology and mandatory items for the 2013/14 survey.

[bib17] Davis L., Barnes A.J., Gross A.C., Ryder J.R., Shlafer R.J. (2019). Adverse childhood experiences and weight status among adolescents. The Journal of Pediatrics.

[bib18] van Dijk R., van der Valk I.E., Dekovic M., Branje S. (2020). A meta-analysis on interparental conflict, parenting, and child adjustment in divorced families: Examining mediation using meta-analytic structural equation models. Clinical Psychology Review.

[bib19] Duriancik D.M., Goff C.R. (2019). Children of single-parent households are at a higher risk of obesity: A systematic review. Journal of Child Health Care.

[bib20] Eidsdottir S., Kristjansson A., Sigfusdottir I.D., Garber C.E., Allegrante J.P. (2013). Secular trends in overweight and obesity among Icelandic adolescents: Do parental education levels and family structure play a part?. Scandinavian Journal of Public Health.

[bib21] Elfhag K., Rasmussen F. (2008). Food consumption, eating behaviour and self-esteem among single v. married and cohabiting mothers and their 12-year-old children. Public Health Nutrition.

[bib22] Fatima Y., Doi S.A., Mamun A.A. (2015). Longitudinal impact of sleep on overweight and obesity in children and adolescents: A systematic review and bias-adjusted meta-analysis. Obesity Reviews.

[bib23] Fismen A.S., Smith O.R.F., Samdal O., Helleve A., Haug E. (2020). Associations between family structure and adolescents' food habits. Public Health Nutrition.

[bib24] Flouri E., Midouhas E., Ruddy A. (2016). Socio-economic status and family structure differences in early trajectories of child adjustment: Individual and neighbourhood effects. Health & Place.

[bib25] Franko D.L., Thompson D., Bauserman R., Affenito S.G., Striegel-Moore R.H., National Heart L. (2008). What's love got to do with it? Family cohesion and healthy eating behaviors in adolescent girls. International Journal of Eating Disorders.

[bib26] Fuller A.B., Byrne R.A., Golley R.K., Trost S.G. (2019). Supporting healthy lifestyle behaviours in families attending community playgroups: Parents' perceptions of facilitators and barriers. BMC Public Health.

[bib27] Gibson M.T.H., Banas K., Lutje V., McKee M.J., Martin S.P., Fenton C., Bambra C., Bond L. (2018). Welfare-to-work interventions and their effects on the mental and physical health of lone parents and their children. Cochrane Database of Systematic Reviews.

[bib28] Glaser K., Stuchbury R., Price D., Di Gessa G., Ribe E., Tinker A. (2018). Trends in the prevalence of grandparents living with grandchild(ren) in selected European countries and the United States. European Journal of Ageing.

[bib29] Global B.M.I.M.C., Di Angelantonio E., Bhupathiraju S.N., Wormser D., Gao P., Kaptoge S. (2016). Body-mass index and all-cause mortality: Individual-participant-data meta-analysis of 239 prospective studies in four continents. Lancet.

[bib30] Goodman E., Hinden B.R., Khandelwal S. (2000). Accuracy of teen and parental reports of obesity and body mass index. Pediatrics.

[bib31] Gray L.A., Hernandez Alava M., Kelly M.P., Campbell M.J. (2018). Family lifestyle dynamics and childhood obesity: Evidence from the millennium cohort study. BMC Public Health.

[bib32] Hartley J.E., Levin K., Currie C. (2016). A new version of the HBSC family affluence scale - FAS III: Scottish qualitative findings from the international FAS development study. Child Indicators Research.

[bib34] Herke M., Knochelmann A., Richter M. (2020). Health and well-being of adolescents in different family structures in Germany and the importance of family climate. International Journal of Environmental Research and Public Health.

[bib35] Hobza V., Hamrik Z., Bucksch J., De Clercq B. (2017). The family affluence scale as an indicator for socioeconomic status: Validation on regional income differences in the Czech Republic. International Journal of Environmental Research and Public Health.

[bib36] Hofferth S.L. (2006). Residential father family type and child well-being: Investment versus selection. Demography.

[bib37] Inchley J CD, Budisavljevic S, Torsheim T, Jåstad A, Cosma A et al., editors. Spotlight on adolescent health and well-being. Findings from the 2017/2018 health behaviour in school-aged children (HBSC) survey in Europe and Canada. International report. Volume 1. Key findings. Copenhagen: WHO Regional Office for Europe; 2020. Contract No.: Licence: CC BY-NC-SA 3.0 IGO.

[bib38] Inchley J CD, Young T et al. (editors). Growing up unequal: Gender and socioeconomic differences in young People‘s health and well-being. Health behaviour in school-aged children (HBSC) study: International report from the 2013/2014 survey.Copenhagen: World.

[bib39] Jang M., Owen B., Lauver D.R. (2019). Different types of parental stress and childhood obesity: A systematic review of observational studies. Obesity Reviews.

[bib40] Khadaroo A.M.F. (2021). Parenting of adolescent single children: A mixed-methods study. Journal of Family Issues.

[bib41] Langoy A., Smith O.R.F., Wold B., Samdal O., Haug E.M. (2019). Associations between family structure and young people's physical activity and screen time behaviors. BMC Public Health.

[bib42] Larson N., MacLehose R., Fulkerson J.A., Berge J.M., Story M., Neumark-Sztainer D. (2013). Eating breakfast and dinner together as a family: Associations with sociodemographic characteristics and implications for diet quality and weight status. Journal of the Academy of Nutrition and Dietetics.

[bib43] Lazzeri G., Ahluwalia N., Niclasen B., Pammolli A., Vereecken C., Rasmussen M. (2016). Trends from 2002 to 2010 in daily breakfast consumption and its socio-demographic correlates in adolescents across 31 countries participating in the HBSC study. PLoS One.

[bib44] Levin K.A., Kirby J., Currie C. (2012). Adolescent risk behaviours and mealtime routines: Does family meal frequency alter the association between family structure and risk behaviour?. Health Education Research.

[bib45] Li B., Adab P., Cheng K.K. (2015). The role of grandparents in childhood obesity in China - evidence from a mixed methods study. International Journal of Behavioral Nutrition and Physical Activity.

[bib46] Lobstein T., Baur L., Uauy R., TaskForce I.I.O. (2004). Obesity in children and young people: A crisis in public health. Obesity Reviews.

[bib47] Meller F.O., Loret de Mola C., Assuncao M.C.F., Schafer A.A., Dahly D.L., Barros F.C. (2018). Birth order and number of siblings and their association with overweight and obesity: A systematic review and meta-analysis. Nutrition Reviews.

[bib48] Milanović et al. Social inequalities in physical activity, screen time and sleep patterns among 6-9-year-old children from 24 countries in the WHO European Region. Obesity Review THIS ISSUE.

[bib49] Möhring K. The fxed effects approach as alternative to multilevel models for cross-national analyses GK SOCLIFE Working Paper Series WP 16/2012. 2012;Cologne: University of Cologne.

[bib50] Moncrief T., Beck A.F., Simmons J.M., Huang B., Kahn R.S. (2014). Single parent households and increased child asthma morbidity. Journal of Asthma.

[bib51] Nunes-Costa R.A., Lamela D.J., Figueiredo B.F. (2009). Psychosocial adjustment and physical health in children of divorce. J Pediatr (Rio J)..

[bib53] Panter J., Tanggaard Andersen P., Aro A.R., Samara A. (2018). Obesity prevention: A systematic review of setting-based interventions from nordic countries and The Netherlands. Journal of Obesity.

[bib54] Parikka S., Maki P., Levalahti E., Lehtinen-Jacks S., Martelin T., Laatikainen T. (2015). Associations between parental BMI, socioeconomic factors, family structure and overweight in Finnish children: A path model approach. BMC Public Health.

[bib55] Parlament E. (2020). The situation of single parents in the EU. https://www.europarl.europa.eu/RegData/etudes/STUD/2020/659870/IPOL_STU(2020)659870_EN.pdf.

[bib56] Pearce L.D., Hayward G.M., Chassin L., Curran P.J. (2018). The increasing diversity and complexity of family structures for adolescents. Journal of Research on Adolescence.

[bib57] Pearson N., Atkin A.J., Biddle S.J., Gorely T., Edwardson C. (2010). Parenting styles, family structure and adolescent dietary behaviour. Public Health Nutrition.

[bib58] Perez A., Gabriel K., Nehme E.K., Mandell D.J., Hoelscher D.M. (2015). Measuring the bias, precision, accuracy, and validity of self-reported height and weight in assessing overweight and obesity status among adolescents using a surveillance system. International Journal of Behavioral Nutrition and Physical Activity.

[bib59] Renzaho A.M., Dau A., Cyril S., Ayala G.X. (2014). The influence of family functioning on the consumption of unhealthy foods and beverages among 1- to 12-y-old children in Victoria, Australia. Nutrition.

[bib60] Report C.B. (2016). The majority of children live with two parents. https://www.census.gov/library/stories/2017/08/majority-of-children-live-with-two-parents.html.

[bib61] Sallis J.F., Owen N., Fisher E.B., Glanz K., Rimer B.K., Viswanath K. (2008). Health behavior and health education.

[bib62] Savona N., Macauley T., Aguiar A., Banik A., Boberska M., Brock J. (2021). Identifying the views of adolescents in five European countries on the drivers of obesity using group model building. The European Journal of Public Health.

[bib63] Schaan V.K., Vogele C. (2016). Resilience and rejection sensitivity mediate long-term outcomes of parental divorce. European Child & Adolescent Psychiatry.

[bib64] Sharif M.Z., Alcala H.E., Albert S.L., Fischer H. (2017). Deconstructing family meals: Do family structure, gender and employment status influence the odds of having a family meal?. Appetite.

[bib65] Shloim N., Edelson L.R., Martin N., Hetherington M.M. (2015). Parenting styles, feeding styles, feeding practices, and weight status in 4-12 Year-old children: A systematic review of the literature. Frontiers in Psychology.

[bib66] Sommer A., Twig G. (2018). The impact of childhood and adolescent obesity on cardiovascular risk in adulthood: A systematic review. Current Diabetes Reports.

[bib67] Spencer E.A., Appleby P.N., Davey G.K., Key T.J. (2002). Validity of self-reported height and weight in 4808 EPIC-Oxford participants. Public Health Nutrition.

[bib68] Stahlmann K., Hebestreit A., DeHenauw S., Hunsberger M., Kaprio J., Lissner L. (2020). A cross-sectional study of obesogenic behaviours and family rules according to family structure in European children. International Journal of Behavioral Nutrition and Physical Activity.

[bib69] Steinbach A. (2019). Children's and parents' well-being in joint physical custody: A literature review. Family Process.

[bib70] Stewart S.D., Menning C.L. (2009). Family structure, nonresident father involvement, and adolescent eating patterns. Journal of Adolescent Health.

[bib71] Tabak I., Oblacinska A., Jodkowska M., Mikiel-Kostyra K. (2012). [Changes in structure and socioeconomic position of the family as determinants of overweight in adolescents]. Pediatric Endocrinology, Diabetes and Metabolism.

[bib72] Thomas R., Siliquini R., Hillegers M.H., Jansen P.W. (2020). The association of adverse life events with children's emotional overeating and restrained eating in a population-based cohort. International Journal of Eating Disorders.

[bib73] Thomson E., McLanahan S.S. (2012). Reflections on "Family structure and child well-being: Economic resources vs. Parental socialization. Social Forces.

[bib74] TM P. (2012). The influence of family structure Vs. Family climate on adolescent well-being. Child and Adolescent Social Work Journal.

[bib75] Troxel W.M., Lee L., Hall M., Matthews K.A. (2014). Single-parent family structure and sleep problems in black and white adolescents. Sleep Medicine.

[bib76] Union E. (2020).

[bib77] Vazquez C.E., Cubbin C. (2020). Socioeconomic status and childhood obesity: A review of literature from the past decade to inform intervention research. Current Obesity Reports.

[bib78] Weihrauch-Bluher S., Schwarz P., Klusmann J.H. (2019). Childhood obesity: Increased risk for cardiometabolic disease and cancer in adulthood. Metabolism.

[bib52] WHO (2014).

[bib79] Youngblut J.M., Brooten D., Lobar S.L., Hernandez L., McKenry M. (2005). Child care use by low-income single mothers of preschoolers born preterm versus those of preschoolers born full term. Journal of Pediatric Nursing.

[bib80] Zaborskis A., Grincaite M., Kavaliauskiene A., Tesler R. (2020). Family structure and affluence in adolescent eating behaviour: A cross-national study in forty-one countries. Public Health Nutrition.

